# Thymidine catabolism promotes NADPH oxidase-derived reactive oxygen species (ROS) signalling in KB and yumoto cells

**DOI:** 10.1038/s41598-018-25189-y

**Published:** 2018-04-30

**Authors:** Sho Tabata, Masatatsu Yamamoto, Hisatsugu Goto, Akiyoshi Hirayama, Maki Ohishi, Takuya Kuramoto, Atsushi Mitsuhashi, Ryuji Ikeda, Misako Haraguchi, Kohichi Kawahara, Yoshinari Shinsato, Kentaro Minami, Atsuro Saijo, Yuko Toyoda, Masaki Hanibuchi, Yasuhiko Nishioka, Saburo Sone, Hiroyasu Esumi, Masaru Tomita, Tomoyoshi Soga, Tatsuhiko Furukawa, Shin-ichi Akiyama

**Affiliations:** 1 Institute for Advanced Biosciences, Keio University, 246-2 Mizukami, Kakuganji, Tsuruoka, Yamagata 997-0052 Japan; 2 Department of Molecular Oncology, Graduate School Medical and Dental Sciences, Kagoshima University, 8-35-1 Sakuragaoka, Kagoshima, 890-8544 Japan; 3 Department of Respiratory Medicine and Rheumatology, Institute of Biomedical Sciences, Tokushima University Graduate School, 3-18-15 Kuramoto-cho, Tokushima, 770-8503 Japan; 4 Department of Pharmacy, University of Miyazaki Hospital, 5200 Kihara, Kiyotake-cho, Miyazaki, 889-1692 Japan; 5 Department of Biochemistry and Molecular Biology, Graduate School Medical and Dental Sciences, Kagoshima University, 8-35-1 Sakuragaoka, Kagoshima, 890-8544 Japan; 6 Clinical Research, Research Institute for Biomedical Sciences, Tokyo University of Science, 2641 Yamazaki, Noda, Chiba, 278-0022 Japan; 7 Clinical Research Center, National Kyushu Cancer Center, 3-1-1 Notame Minami-ku, Fukuoka, 811-1395 Japan

## Abstract

Thymidine phosphorylase (TP) is a rate-limiting enzyme in the thymidine catabolic pathway. TP is identical to platelet-derived endothelial cell growth factor and contributes to tumour angiogenesis. TP induces the generation of reactive oxygen species (ROS) and enhances the expression of oxidative stress-responsive genes, such as interleukin (IL)-8. However, the mechanism underlying ROS induction by TP remains unclear. In the present study, we demonstrated that TP promotes NADPH oxidase-derived ROS signalling in cancer cells. NADPH oxidase inhibition using apocynin or small interfering RNAs (siRNAs) abrogated the induction of *IL-8* and ROS in TP-expressing cancer cells. Meanwhile, thymidine catabolism induced by TP increased the levels of NADPH and intermediates of the pentose phosphate pathway (PPP). Both siRNA knockdown of glucose 6-phosphate dehydrogenase (G6PD), a rate-limiting enzyme in PPP, and a G6PD inhibitor, dihydroepiandrosterone, reduced TP-induced ROS production. siRNA downregulation of 2-deoxy-D-ribose 5-phosphate (DR5P) aldolase, which is needed for DR5P to enter glycolysis, also suppressed the induction of NADPH and *IL-8* in TP-expressing cells. These results suggested that TP-mediated thymidine catabolism increases the intracellular NADPH level via the PPP, which enhances the production of ROS by NADPH oxidase and activates its downstream signalling.

## Introduction

Angiogenesis is a critical determinant of tumour growth and metastasis, as well as of wound healing, embryonic development, and arteriosclerosis^1^. We previously demonstrated that thymidine phosphorylase (TP), an enzyme that converts thymidine to thymine and 2-deoxy-D-ribose 1-phosphate, is identical to platelet-derived endothelial cell growth factor (PD-ECGF), an angiogenic factor^2^, and that 2-deoxy-D-ribose (DR), a degradation product of thymidine produced by TP, also presents angiogenic activity^3,4^.

Previous findings indicate that TP is overexpressed in various cancer types and contributes to tumour angiogenesis^3,5^. The enzymatic activity of TP is essential for angiogenesis by TP^6^. Considering that inhibitors of TP activity could suppress angiogenesis, and consequently tumour growth and metastasis in TP-expressing tumours, we developed a novel selective inhibitor of TP (TPI; 5-chloro-6-(2-iminopyrrolidin-1-yl) methyl-2,4 (1 H,3 H)-pyrimidinedione hydrochloride; Ki = 2 × 10^−8^ M)^7^, which has several advantages over previously described inhibitors of TP. Using this TPI, we demonstrated that TP plays a key role in the invasiveness and metastasis of TP-expressing solid tumours, and suggested that TPI may be an anti-metastatic agent for blood-borne metastasis^8^.

Both TP and DR are involved in the production of reactive oxygen species (ROS) and increase the secretion of stress-induced angiogenic cytokines, such as vascular endothelial growth factor (VEGF), matrix metalloproteinase (MMP)-1, and interleukin (IL)-8^5,9,10^. TP is expressed in numerous tumours; therefore, it may contribute to the progression of malignant tumours by generating oxidative stress^3^. Recently, we found that TP-mediated thymidine catabolism could supply the carbon source in glycolysis and the pentose phosphate pathway (PPP), thus contributing to cell survival under nutrient starvation^11^. However, the relationship between thymidine catabolism and oxidative stress remains unclear. In this study, we investigated the molecular basis for ROS generation by TP.

## Results

### Role of TP in ROS generation and IL-8 expression in human cancer KB cells

Previous findings suggested that TP induces oxidative stress and consequently enhances the expression of angiogenic factors, VEGF, MMP-1, and IL-8 in TP-overexpressing cells^5,9,10^. We also reported that the mRNA and protein levels of *IL-8* in TP-overexpressing human epidermoid carcinoma KB (KB/TP) cells were higher than those in KB (KB/CV) cells that were transfected with control vector (CV)^12^.

**Figure 1:**
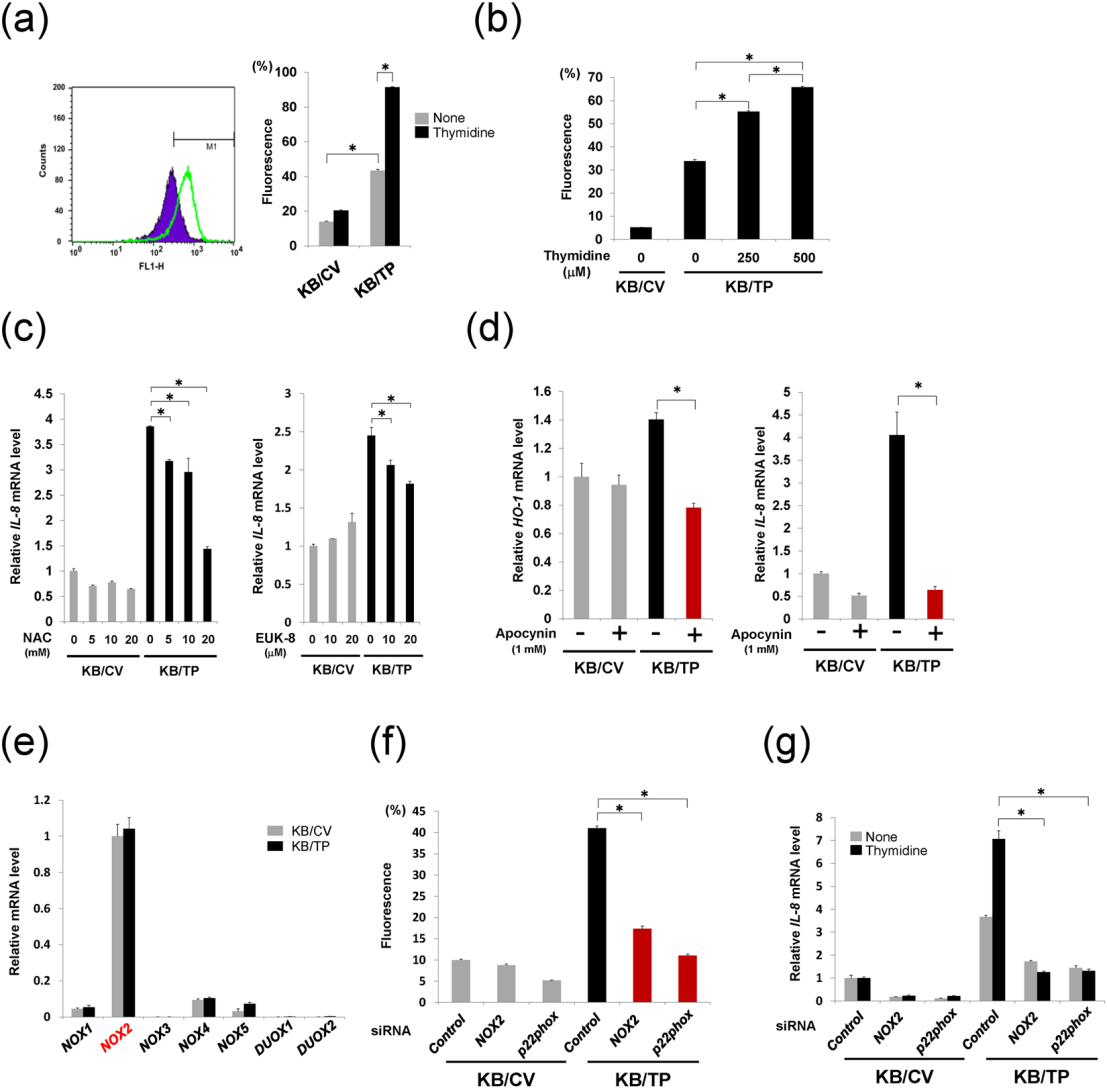
Involvement of NADPH oxidase in TP-induced ROS generation. (**a**) A representative sample for three independent FACS analyses is displayed (Left). Cells were treated with 500 μM thymidine for 48 h, and then ROS levels were measured in the cells. Proportions of cells in the M1 fraction are shown as relative levels of ROS. Relative ROS levels in KB/CV and KB/TP cells in the absence or presence of 500 μM thymidine are measured by using H_2_DCF-DA fluorescence dye (Right). (**b**) ROS levels in KB/CV and KB/TP cells incubated in the absence or presence of thymidine at 250 or 500 μM for 48 h. (**c**) Attenuated expression of *IL-8* mRNA in KB/TP cells treated with NAC (left) and EUK8 (right) for 48 h. Expression of *IL-8* in KB/CV and KB/TP cells was determined by real-time PCR. (**d**) Decreased expression of *HO-1* and *IL-8* mRNAs in KB/TP cells treated with apocynin for 48 h. (**e**) Expression of NOX isoforms in KB cells. Expression levels of *NOX* isoforms in KB/CV and KB/TP cells were determined by real-time PCR. (**f**) The effect of down-regulation of *NOX2* and *p22phox* on the production of ROS in KB/CV and KB/TP cells. The cells transfected with *NOX2* siRNA or *p22phox* siRNA were treated with 10 μM H_2_DCF-DA for 1 h and ROS levels were determined by using FACScan. (**g**) KB cells transfected with *NOX2* siRNA or *p22phox* siRNA were treated with or without 500 μM thymidine for 48 h and *IL-8* mRNA expression levels were determined by real-time PCR. Data are presented as mean ± SD. ^∗^*P* < 0.01.

First, we examined TP-induced ROS production and found that ROS levels in the absence and presence of 500 μM thymidine in KB/TP cells were 3.1- and 4.4-fold higher than those in KB/CV cells, respectively (Fig. 1a). The ROS level increased with increasing concentrations of thymidine in KB/TP cells (Fig. 1b). To confirm whether TP-mediated ROS induces the expression of *IL-8*, KB/CV and KB/TP cells were treated with various concentrations of antioxidants, N-acetyl-L-cysteine (NAC) and EUK-8. *IL-8* mRNA expression was higher in KB/TP cells than in KB/CV cells, and NAC and EUK-8 suppressed the enhanced expression of *IL-8* mRNA in KB/TP cells in a dose-dependent manner (Fig. 1c). These results indicated that the ROS generated in KB/TP cells enhanced *IL-8* mRNA transcription.

### Role of NADPH oxidase in ROS generation by TP

ROS generated by NADPH oxidase stimulates diverse redox signalling pathways leading to angiogenesis and cell growth^13,14^. Therefore, we examined the effect of apocynin, an inhibitor of NADPH oxidase, on increased expression of *heme oxygenase 1* (*HO-1*), a cellular oxidative stress marker, and *IL-8* in KB/TP cells. Apocynin, at a concentration of 1 mM, suppressed the enhanced expression of *HO-1* mRNA in KB/TP cells (Fig. 1d, left), suggesting that the augmented ROS level in KB/TP cells is generated by NADPH oxidase. Apocynin also suppressed *IL-8* mRNA expression in KB/TP cells to the level observed in KB/CV cells (Fig. 1d, right).

**Figure 2:**
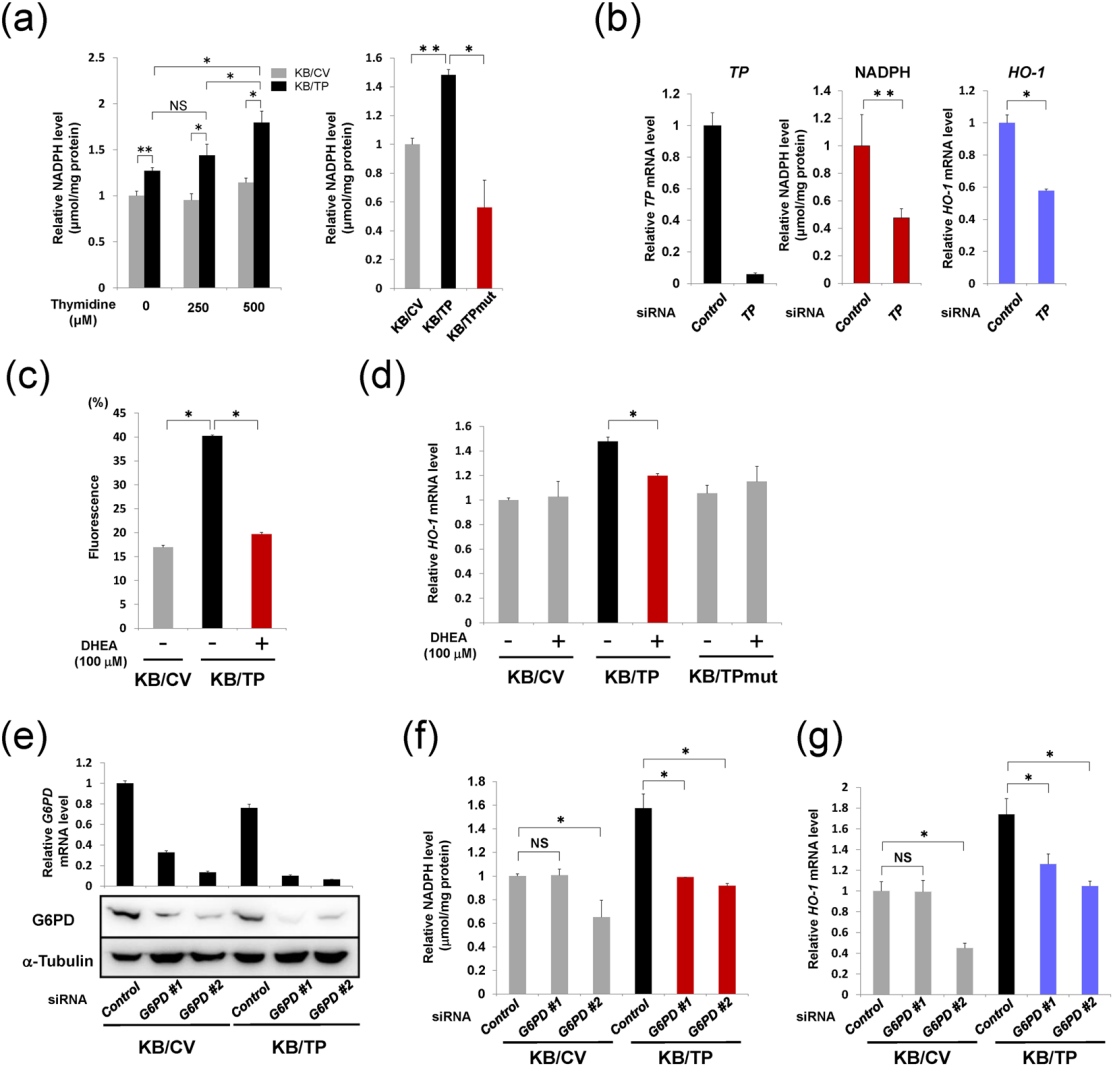
Effect of TP on Intracellular levels of NADPH (**a**) NADPH levels in KB/CV and KB/TP cells incubated in the absence or presence of thymidine at 250 or 500 μM for 48 h (left). Effect of TP activity on NADPH levels in KB cells. NADPH levels in KB/CV, KB/TP and KB/TPmut cells treated with or without 500 μM thymidine for 48 h were determined (right). NADPH levels in KB/CV and KB/TP cells were measured using a NADPH assay kit. (**b**) Effect of TP knockdown on *TP* mRNA (left), NADPH (middle), and *HO-1* mRNA (right) levels in Yumoto cells. (**c**) ROS levels in KB/TP cells treated with DHEA for 24 h. (**d**) Expression levels of *HO-1* mRNA were determined by real-time PCR in KB/CV, KB/TP, and KB/TPmut cells treated with DHEA for 48 h. (**e**) KB cells were transfected with *G6PD* siRNA or negative control siRNA. The levels of *G6PD* mRNA (upper) and G6PD protein (lower) were determined by real-time PCR and immunoblotting, respectively. (**f**) Effect of downregulation of G6PD on NADPH levels in KB cells. KB cells transfected with *G6PD* siRNAs were treated with 500 μM thymidine for 48 h, and NADPH intracellular levels were determined. (**g**) Effect of downregulation of G6PD on *HO-1* mRNA levels in KB cells. Data are presented as mean ± SD. ^∗^*P* < 0.01, ^∗∗^*P* < 0.05.

Seven members of the NADPH oxidase family have been identified in mammalian species, each of which includes a different membrane-spanning catalytic subunit, NOX 1–5 and DUOX1 and 2^15,16^. *NOX1, 2, 4* and 5 mRNAs were expressed, but *NOX3, DUOX1*, and *DUOX2* mRNA expression was marginal in KB/CV and KB/TP cells (Fig. 1e). Meanwhile, apocynin has been reported to inhibit NOX2-dependent superoxide production^17^. Therefore, we prepared siRNAs for *NOX2* and *p22phox*, which are essential components of NOX2-containing NADPH oxidase, and down-regulated these genes to confirm the effect of apocynin. *NOX2* and *p22phox* mRNA and protein expression levels were efficiently decreased by the siRNAs targeting *NOX2* and *p22phox*, respectively (Supplementary Fig. S1). ROS levels in KB/TP cells were significantly decreased by downregulation of *NOX2* and *p22phox* (Fig. 1f). The expression levels of *IL-8* mRNA in KB/TP cells in the absence and presence of thymidine were also considerably decreased by *NOX2* and *p22phox* siRNAs (Fig. 1g). These results indicated that NOX2-containing NADPH oxidase is involved in ROS production in KB/TP cells. In addition to KB cells, we examined human cervical cancer Yumoto cells, which intrinsically express TP. We previously reported that TPI suppressed ROS level and *IL-8* expression in Yumoto cells^12^. NAC also abrogated *IL-8* expression in Yumoto cells^12^. The expression levels of NADPH oxidase isoforms in Yumoto cells were different from those in KB cells (Supplementary Fig. S2a). *DUOX1* expression in Yumoto cells was higher than that in KB cells. Down-regulation of TP by a *TP-*targeting siRNA (Fig. 2b, left) suppressed the expression of *IL-8*, but not that of *DUOX1* in Yumoto cells (Supplementary Fig. S2b). Furthermore, the attenuation of DUOX1 using a siRNA (Supplementary Fig. S2c) decreased the ROS levels (Supplementary Fig. S2d) and *IL-8* expression (Supplementary Fig. S2e) in Yumoto cells. These data suggested that, as well as the TP-NOX2 signalling in KB cells, DUOX1 increased the levels of ROS and *IL-8* in Yumoto cells.

### Effect of TP on intracellular levels of NADPH

Levels of NADPH, a substrate for NADPH oxidase, increased with increasing concentrations of thymidine in KB/TP cells (Fig. 2a, left). NADPH levels in KB/TP cells were higher than those in KB/CV cells and in KB (KB/TPmut) cells, which express an enzymatically inactive mutant TP (Fig. 2a, right). These findings suggested that NADPH levels were increased by TP, and that the enzyme activity is required for increased production of NADPH. Treatment of Yumoto cells with *TP* siRNA considerably attenuated the expression of TP (Fig. 2b, left), and decreased the levels of NADPH and *HO-1* mRNA to 67 and 57% of those in control cells, respectively (Fig. 2b, middle and right).

NADPH is mainly generated during the oxidative phase of the PPP. Previous reports demonstrated that the suppression of glucose 6-phosphate dehydrogenase (G6PD), the first and rate-limiting enzyme of the PPP, attenuated NADPH oxidase-derived ROS generation in different cell types^18–21^. The expression levels of G6PD in KB/TP cells were slightly lower than those in KB/CV cells (Fig. 2e). We examined the effect of dihydroepiandrosterone (DHEA), an inhibitor of G6PD, on the enhanced ROS production in KB/TP cells. DHEA suppressed the levels of ROS (Fig. 2c) and *HO-1* mRNA expression (Fig. 2d) in KB/TP cells, but did not affect the expression of *HO-1* in KB/CV and KB/TPmut cells (Fig. 2d). DHEA also decreased *HO-1* expression in Yumoto cells (Fig. S2f). Furthermore, when the expression of G6PD was down-regulated in KB/CV and KB/TP cells (Fig. 2e), the levels of NADPH (Fig. 2f) and *HO-1* mRNA expression (Fig. 2g) in KB/TP cells decreased. These results suggested that TP augmented NADPH levels through the oxidative phase of the PPP, which increased the generation of NADPH oxidase-derived ROS.

NADPH enhances NADPH oxidase-derived ROS generation, whereas it augments reduced glutathione (GSH) levels and suppresses the oxidative status in cells. We previously reported that expression of γ-glutamylcysteine synthetase (γ-GCS), a rate-limiting enzyme of glutathione synthesis, and the total glutathione level in KB/TP cells were significantly lower than those in KB/CV and KB/TPmut cells^12^. In this study, we evaluated the GSH level in KB/TP cells and found it to be lower than that in KB/CV cells, regardless of the NADPH levels in the cells (Supplementary Fig. S3). These results suggested that the generation of GSH is attenuated in KB/TP cells.

### Augmentation of PPP metabolites in KB/TP cells

**Figure 3:**
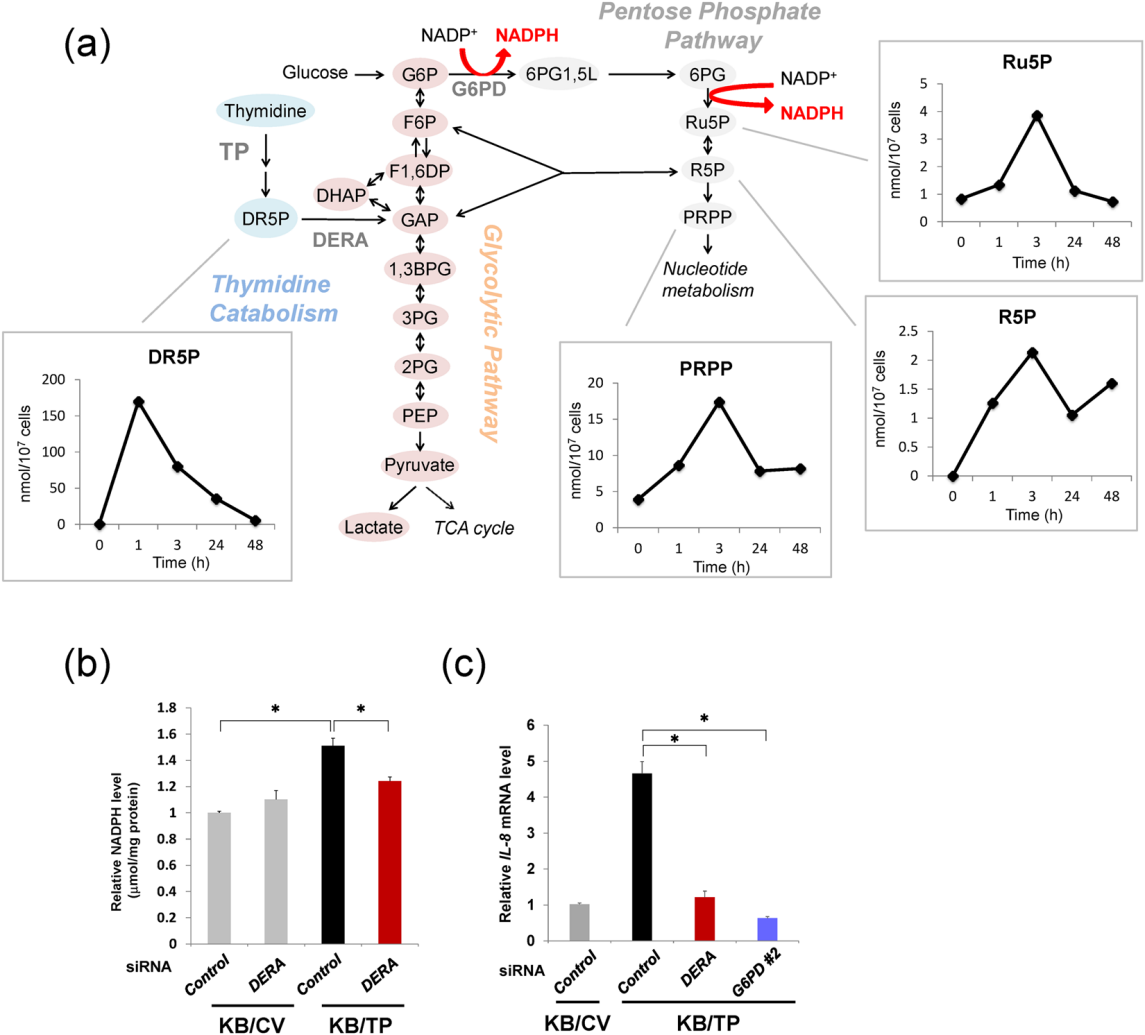
Levels of PPP intermediates in KB/TP cells after addition of thymidine. (**a**) KB/TP were incubated in serum-free medium with 500 μM thymidine for the indicated times, and levels of metabolites were determined using CE-TOFMS. (**b**) KB cells transfected with DERA siRNA were treated with 500 μM thymidine for 48 h and the intracellular levels of NADPH were determined. (**c**) Effect of DERA and G6PD downregulation on *IL-8* mRNA levels in KB/TP cells. Data are presented as mean ± SD. ^∗^*P* < 0.01.

**Figure 4:**
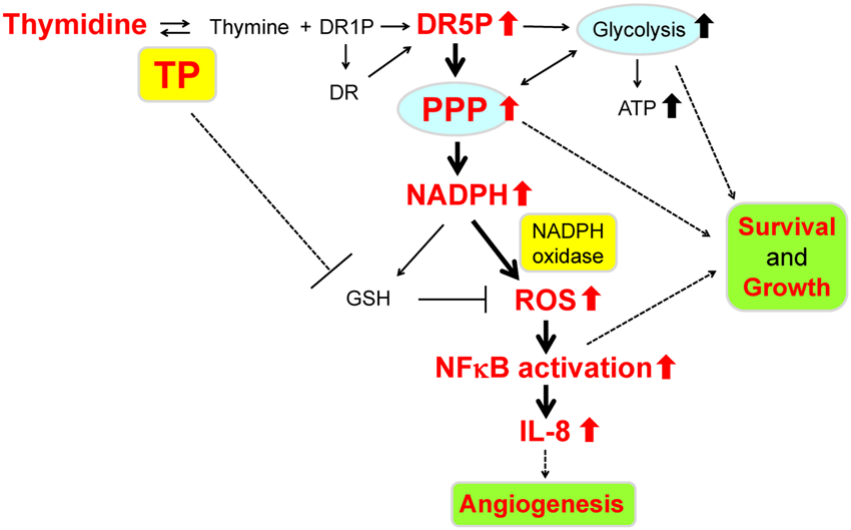
Schematic representation of the molecular mechanism underlying TP-induced ROS production.

We previously reported that thymidine catabolism by TP can supply the carbon source in the glycolytic pathway and the PPP^11^. Figure 3a shows the catabolic pathway of thymidine. We investigated time-dependent changes in metabolite levels of the PPP in KB/TP cells using capillary electrophoresis time-of-flight mass spectrometry (CE-TOFMS) after the addition of thymidine. Intracellular levels of thymidine-derived DR5P increased and achieved peak levels 1 h after the addition of thymidine (Fig. 3a). The levels of PPP metabolites, ribulose-5-phosphate (Ru5P), ribose 5-phosphate (R5P), and 5-phosphoribosyl 1α-diphosphate (PRPP), increased and achieved peaks at 3 h (Fig. 3a). Next, we examined the effect of silencing of 2-deoxy-D-ribose 5-phosphate aldolase (DERA), a key enzyme in the pathway by which thymidine-derived DR5P enters glycolysis and the PPP (Fig. 3a), on the levels of NADPH in KB/TP cells treated with thymidine. *DERA* siRNA suppressed NADPH in KB/TP cells (Fig. 3b). siRNAs against *DERA* and *G6PD* also decreased *IL-8* expression (Fig. 3c). These results suggested that the augmentation of PPP flux by TP-mediated thymidine catabolism generated intracellular NADPH, which consequently was involved in the expression of *IL-8* in KB/TP cells (Fig. 4).

## Discussion

When present in high quantities, ROS induce apoptosis and senescence; however, they function as signalling molecules at lower levels^22^. ROS are generated in many tumours and are involved in tumour angiogenesis^23^. ROS can be generated from several sources, such as the mitochondrial electron transport system, cytochrome p450, uncoupled NO synthase (NOS), xanthine oxidase, and NADPH oxidase. Among them, NADPH oxidase is the only known mammalian enzyme system dedicated to the production of ROS alone. ROS generated by NADPH oxidase participate in intracellular signalling pathways that regulate the proliferation of vascular and non-vascular cells^24,25^. NADPH oxidases have specific subcellular localisations. Spatially confined ROS production in close proximity to redox-sensitive targets may permit ROS to activate specific redox signalling pathways. NADPH oxidase-derived ROS were previously suggested to increase cell growth by direct activation of certain oxidative-response transcription factors including nuclear factor kappaB (NFκB)^26^. We also found that ROS generated in TP-expressing KB cells activated canonical NFκB signalling and enhanced the expression of genes regulated by NFκB^27^.

We assumed that TP is involved in some mechanisms for ROS production and their mechanistic dominance differs depending on cell type or nutrient status. Brown *et al*. demonstrated that TP induced expression of an oxidative stress marker HO-1 in human colon carcinoma cells (Colo320 cells) and human urinary bladder carcinoma cells (RT112 cells), and discussed that the DR generated by TP during thymidine catabolism may contribute to ROS production by finally being converted to enediol via schiff base reactions^9^. Additionally, we and other group reported that TP suppresses the synthesis of glutathione, leading to the increase in ROS production^12,28^. In this paper, we also reported that TP contributed to enhance ROS level via NADPH oxidase.

To examine the effect of thymidine in KB/TP cells, the cells were treated with 250 or 500 μM thymidine; the results are shown in Figs 1a,b,g, 2a,f and 3a,b. The levels of thymidine used in the *in vitro* experiments were higher than those in sera. However, in a specific region of the tumour, such as a necrotic area, the thymidine level might be considerably elevated, because DNA of necrotic cells is degraded and supplies a large amount of thymidine^9^. Even in normal cell culture conditions without supplemental thymidine, levels of ROS, HO-1, IL-8, and NADPH in KB/TP were higher than those in KB/CV (Figs 1a,d and 2a). Therefore, we consider that TP signalling functions at physiological thymidine concentrations.

NADPH has a dual effect, oxidative and anti-oxidative. NADPH is a substrate for NADPH oxidase that generates ROS, whereas it augments GSH, which serves as the intracellular anti-oxidant. The NADPH level in KB/TP cells was higher than that in KB/CV cells, while the GSH level in KB/TP cells was lower than that in KB/CV cells (Supplementary Fig. S3). Our previous findings suggested that TP-mediated thymidine catabolism lead to the decrease in γ-GCS and glutathione levels in KB cells^12^. The decrease in the level of total glutathione in KB/TP cells may be the cause of the low levels of NADPH-induced GSH in KB/TP cells. These results suggest that NADPH in KB/TP cells is preferentially utilised by NADPH oxidase rather than glutathione reductase which generates GSH (Fig. 4).

Alternatively, some papers reported the pro-oxidative effect of NADPH oxidase, which is induced by NFκB transcriptional activation^29–31^. Expression of NADPH oxidase subunit p22phox was increased by NFκB in aortic smooth muscle and pancreatic cancer cells^32,33^. We also demonstrated that TP induced the activation of NFκB in KB and Yumoto cells^27^ and found that expression of *p22phox* in KB/TP cells was higher than that in KB/CV cells (Supplementary Fig. S1). NFκB activation in TP-expressing cells was suppressed by antioxidants^27^, suggesting that the relationship between NADPH oxidase-generated ROS and NFκB activation comprises positive feedback with each other. Further studies are needed to clarify this relationship in TP-expressing cancer cells.

In this study, we demonstrated that TP elevates the intracellular levels of NADPH generated in the PPP, which enhances the production of ROS by NADPH oxidase. ROS consequently induces the expression of IL-8, a pro-angiogenic factor (Fig. 4).

## Methods

### Chemicals and cell culture

NAC was obtained from Sigma-Aldrich (St. Louis, MO, USA). Apocynin and EUK-8 were obtained from Calbiochem (San Diego, CA, USA). 2′,7′-dichlorodihydrofluorescein diacetate (H_2_DCF-DA) was obtained from Molecular Probes (Eugene, OR, USA). Human epidermoid carcinoma KB and human cervical carcinoma Yumoto cells were grown in Dulbecco’s modified Eagle’s medium (DMEM, Nissui Seiyaku, Tokyo, Japan) containing 10% foetal bovine serum, 2 mM glutamine, and 100 units/mL of penicillin at 37 °C in a 5% CO_2_ humidified atmosphere. The medium was changed to fresh serum-free medium before the experiments.

### Transfection of TP/PD-ECGF cDNA into KB cells

We previously established the following stable cell lines: KB/CV, KB/TP, and KB/TPmut^10^. The TP/PD-ECGF full-length cDNA plasmid, TP/PD-ECGF mutant plasmid (L148R, Leu-148→Arg)^6^, or the empty vector was transfected into KB cells by electroporation^34^. After selection with geneticin, the expression of TP in each clone was determined by immunoblotting analysis using an anti-TP monoclonal antibody, as previously described^35^. ATP-positive clone (KB/TP cells) and a control vector-transfected clone (KB/CV cells) were used for further analysis.

### Real-time PCR analysis

Real-time PCR analysis was conducted as previously described^27^. Quantitative measurements were determined using the ^ΔΔ^*Ct* method, and glyceraldehyde-3-phosphate (*GAPDH*) expression was used as the internal control. The primers for real-time PCR are described in Table S1.

### Immunoblotting analysis

Immunoblotting analysis was performed as previously described^12^. Primary antibodies against DUOX1 (Santa Cruz Biotechnology, Santa Cruz, CA, USA), G6PD (Sigma-Aldrich), β-Actin (Santa Cruz Biotechnology), and horseradish peroxidase-conjugated secondary antibodies (GE Healthcare, Buckinghamshire, UK) were used.

### NADPH assay

NADPH levels were determined by using EnzyChrom™ NADP^+^/NADPH assay kit (BioAssay Systems, Hayward, CA, USA) following the manufacturer’s instructions.

### GSH assay

GSH levels were measured by using the Glutathione (GSH/GSSG/Total) Fluorometric Assay Kit (BioVision, Milpitas, CA, USA) following the manufacturer’s instructions.

### RNA interference

*NOX2, p22phox*, and *G6PD* siRNA duplexes were synthesised *in vitro* by a Silencer™ siRNA construction kit (Thermo Fisher Scientific, Waltham, MA, USA) following the manufacturer’s instructions. *TP*, *DERA, DUOX1*, and negative control siRNA duplexes were purchased from Sigma. Depletion of DERA was conducted as previously described^11^. Cells were transfected with the siRNAs using Lipofectamine RNAiMAX (Thermo Fisher Scientific) following the manufacturer’s instructions.

### Measurement of cellular ROS level

ROS production was measured using H_2_DCF-DA, an uncharged cell-permeable fluorescent probe. Cells were treated with H_2_DCF-DA (10 μM), washed, re-suspended in phosphate buffered saline and analysed using FACScan (FACSCalibur, BD Bioscience, San Jose, CA, USA) as described previously^36^.

### Metabolite quantification of KB cells using CE-TOFMS

Intracellular metabolites were measured by CE-TOFMS (Agilent Technologies, Santa Clara, CA, USA) as previously described^37^. The data obtained were analysed using MasterHands^38^. The metabolite identities were determined by matching their *m/z* values and migration times with those of their standard compounds.

### Statistical analysis

Statistical analysis was conducted as previously described^12^. Results were statistically analysed using GraphPad prism v5.0 software. Statistical analyses for all experiments including more than two groups were performed by using one-way analysis of variance (ANOVA). Student’s t tests were used for experiments including two groups. Data are presented as means ± SD. The differences were considered statistically significant at *P* < 0.05.

## Electronic supplementary material

supplemental information
