# Supplementary material for: Thymidine catabolism promotes NADPH oxidase-derived reactive oxygen species (ROS) signalling in KB and yumoto cells
Source: Sci Rep. 2018 Apr 30;8:6760. doi: 10.1038/s41598-018-25189-y (PMC5928239; doi:10.1038/s41598-018-25189-y)
Supplement: Supplementary file 1 — supplemental information [file 41598_2018_25189_MOESM1_ESM.pdf]

Supplemental Information

Thymidine catabolism promotes NADPH oxidase-derived reactive oxygen species (ROS) signalling in KB and yumoto cells

Sho Tabata[1], Masatatsu Yamamoto[2], Hisatsugu Goto[3], Akiyoshi Hirayama[1], Maki Ohishi[1], Takuya Kuramoto[3], Atsushi Mitsuhashi[3], Ryuji Ikeda[4], Misako Haraguchi[5], Kohichi Kawahara[2], Yoshinari Shinsato[2], Kentaro Minami[2], Atsuro Saijo[3], Yuko Toyoda[3], Masaki Hanibuchi[3], Yasuhiko Nishioka[3], Saburo Sone[3], Hiroyasu Esumi[6], Masaru Tomita[1], Tomoyoshi Soga[1], Tatsuhiko Furukawa[2], Shin-ichi Akiyama[7]

[1]Institute for Advanced Biosciences, Keio University, 246-2 Mizukami, Kakuganji, Tsuruoka, Yamagata 997-0052, Japan.

[2]Department of Molecular Oncology, Graduate School Medical and Dental Sciences, Kagoshima University, 8-35-1 Sakuragaoka, Kagoshima 890-8544, Japan.

[3]Department of Respiratory Medicine and Rheumatology, Institute of Biomedical Sciences, Tokushima University Graduate School, 3-18-15 Kuramoto-cho, Tokushima 770-8503, Japan.

[4]Department of Pharmacy, University of Miyazaki Hospital, 5200 Kihara, Kiyotake-cho, Miyazaki, 889-1692, Japan.

[5]Department of Biochemistry and Molecular Biology, Graduate School Medical and Dental Sciences, Kagoshima University, 8-35-1 Sakuragaoka, Kagoshima 890-8544, Japan.

[6]Clinical Research, Research Institute for Biomedical Sciences, Tokyo University of Science, 2641 Yamazaki, Noda, Chiba 278-0022, Japan.

[7]Clinical Research Center, National Kyushu Cancer Center, 3-1-1 Notame Minami-ku, Fukuoka 811-1395, Japan.

SUPPLEMENTAL FIGURES AND TABLE

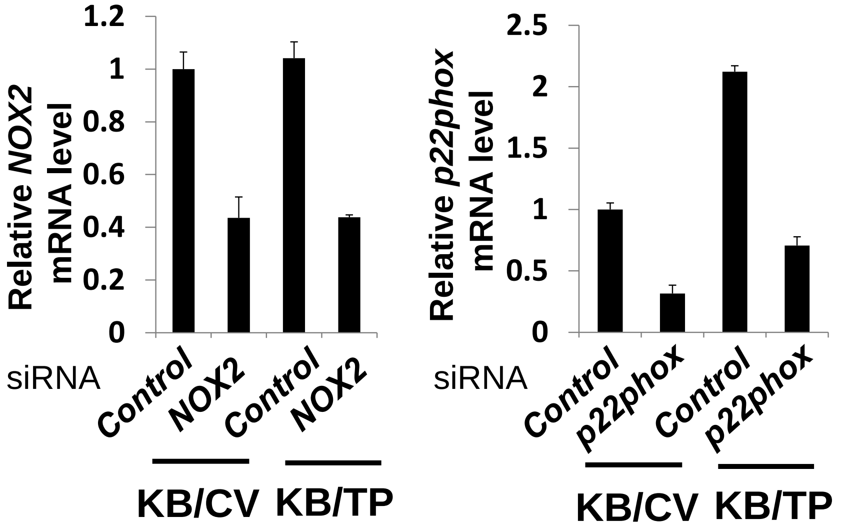

Figure S1. Knock down of *NOX2* and *p22phox* in KB/CV and KB/TP cells. KB/CV and KB/TP cells were transfected with *NOX2* siRNA or *p22phox* siRNA. mRNA levels of NOX2 and p22phox were determined by real time PCR.

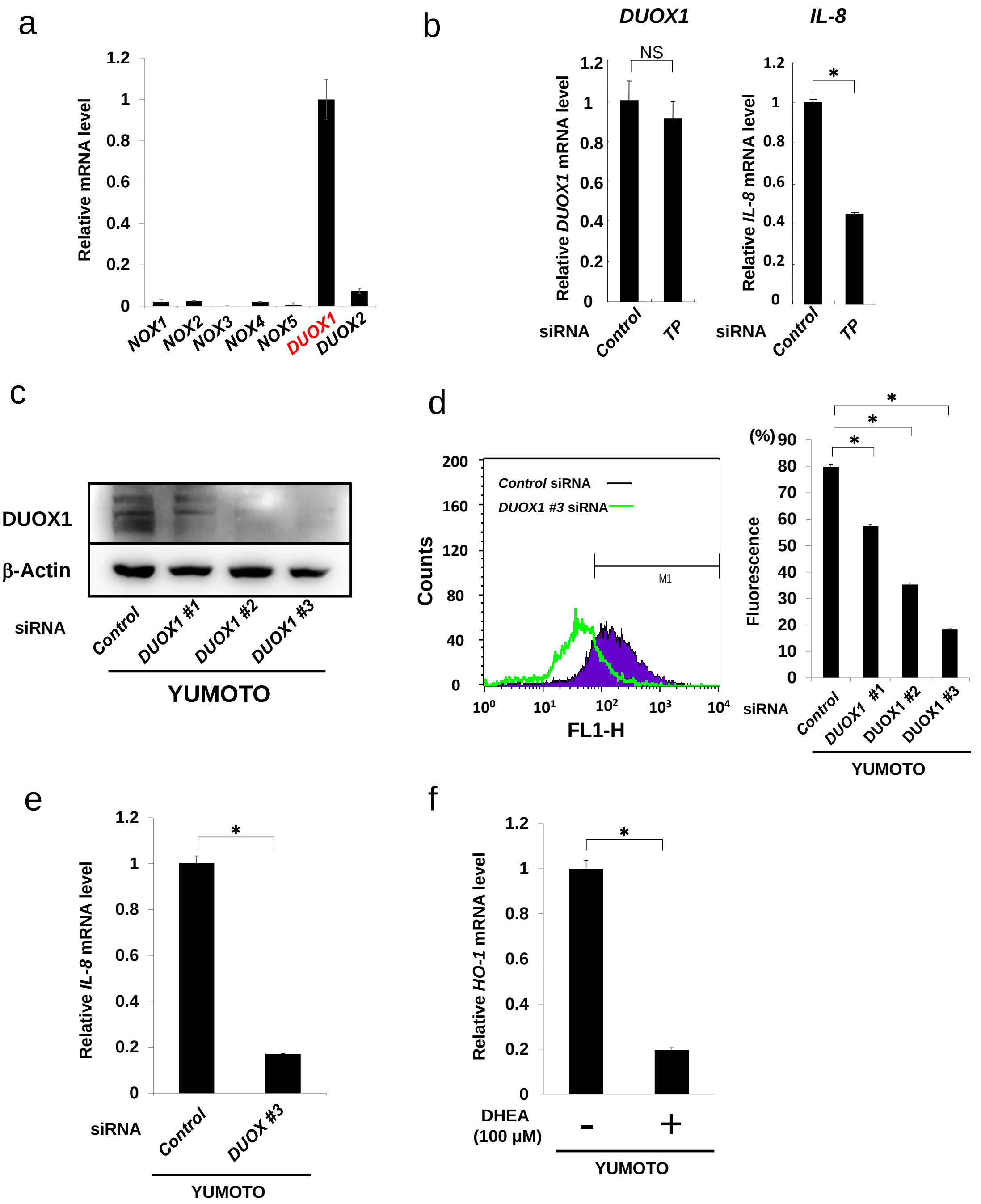

Figure S2. Knockdown of DUOX1 in Yumoto cells. (a) Expression of *NOX* isoforms in Yumoto. Expression levels of *NOX* isoforms in Yumoto cells were determined by real-time PCR. (b) Effect of TP silencing on *DUOX1* and *IL-8* expression in Yumoto cells. (c) Knockdown of DUOX1 in Yumoto cells. (d) Effect of DUOX1 downregulation on the generation of ROS in Yumoto cells. Yumoto cells transfected with *DUOX1* siRNA were treated with 10 μM H_2DCF-DA for 1h and the ROS levels were determined by using FACScan. (d) Effect of DUOX1 knockdown on *IL-8* expression in Yumoto cells. (f) Effect of DHEA on HO-1 expression in Yumoto cells. Data are presented as mean ± SD. *$P < 0.01$.

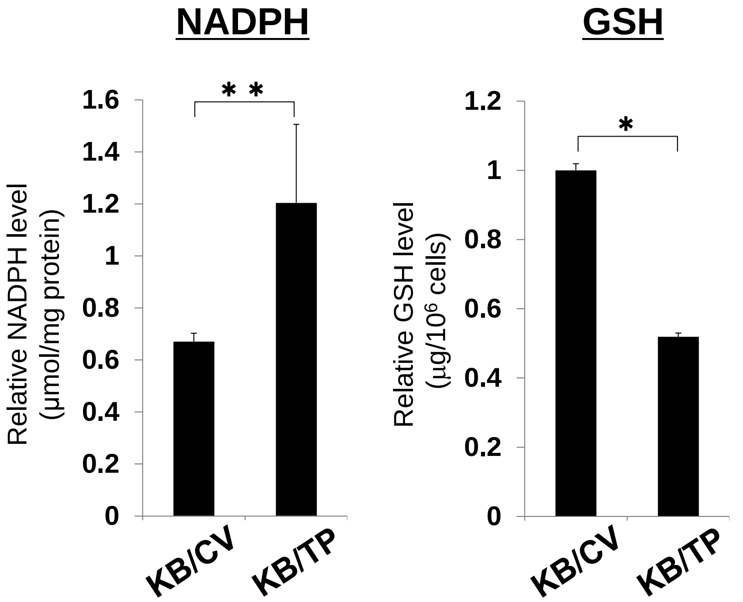

Figure S3. NADPH and GSH levels in KB/CV and KB/TP cells. Effect of TP on levels of NADPH (left) and GSH (right) in KB cells. NADPH levels in KB/CV and KB/TP cells were measured using a NADPH assay kit. GSH levels in KB/CV and KB/TP cells were determined using a GSH assay kit. Data are presented as mean ± SD. **P* < 0.01, ***P* < 0.05.

Table S1. Primer sequences for real-time PCR assays.

Gene	Forward Primer (5′→3′)	Reverse Primer (5′→3′)
TP	GCTGGAGTCTATTCCTGGATTC	ACTGAGAATGGAGGCTGTGATG
IL-8	CCTGATTTCTGCAGCTCTGTGT	GGTGGAAAGGTTTGGAGTATGTCT
HO-1	CGGGCCAGCAACAAAGTGCAAG	GTGTAAGGACCCATCGGAGAAG
NOX1	ACAAATTCCAGTGTGCAGACCA	AGACTGGAATATCGGTGACAGCA
NOX2	CTGCGATTCACACCATTGCAC	CGTGATGACAACTCCAGTGATG
NOX3	ATGCAACCATCCACATCGTG	CGCCTGCTATTGTCCTTAGC
NOX4	CAGAAGGTTCCAAGCAGGAG	AAGTTGAGGGCATTCACCAG
NOX5	CAGCTCTGCATGTGAAAGAG	CATCGATGTCATACACCTGG
DUOX1	CGACATTGAGACTGAGTTGA	CTGGAATGACGTTACCTTCT
DUOX2	AACCTAAGCAGCTCACAACT	CAGAGAGCAATGATGGTGAT
p22phox	TCCTGCATCTCCTGCTCTC	CACAGCCGCCAGTAGGTAG
GAPDH	GTCAACGGATTTGGTCGTAT	TGGTGATGGGATTTCCATTG

- Figure 1E

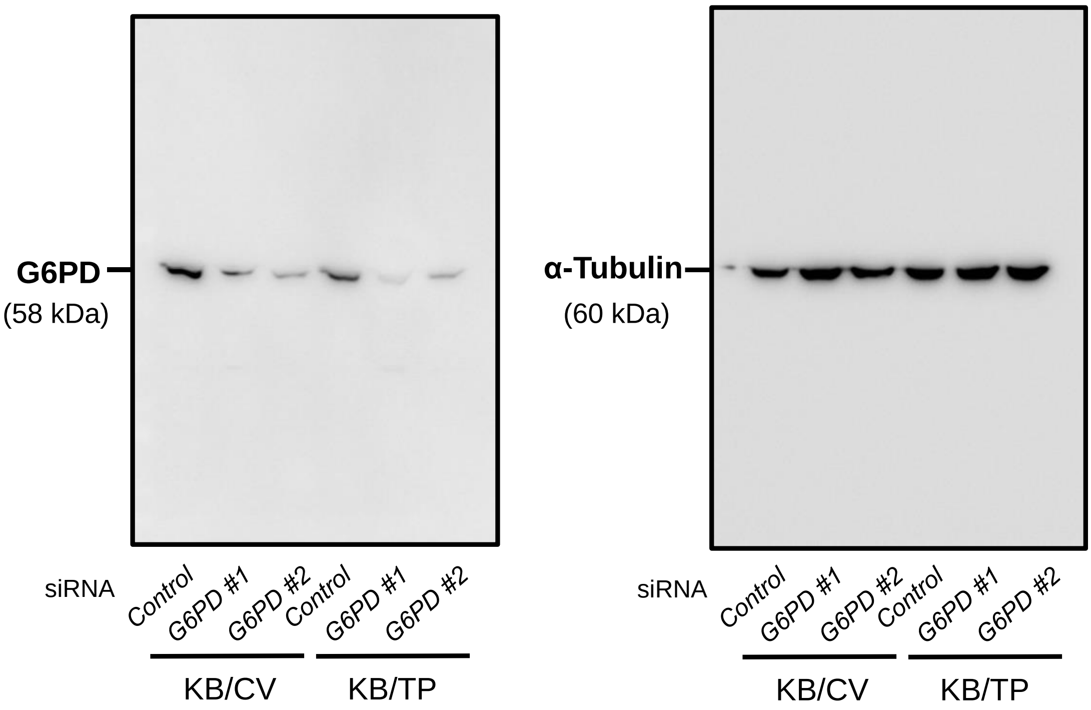

- Figure S2

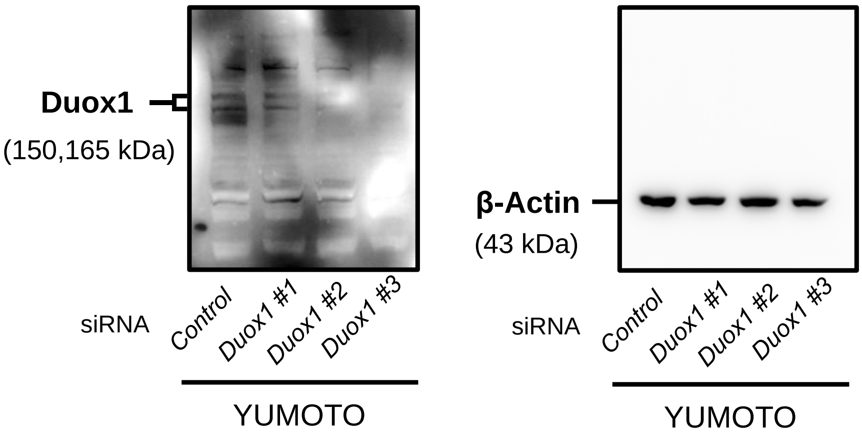

Figure S4. Uncropped images used in Figures 1E and S2.
